# Causes of adult female deaths in Bangladesh: findings from two National Surveys

**DOI:** 10.1186/s12889-015-2256-6

**Published:** 2015-09-18

**Authors:** Quamrun Nahar, Shams El Arifeen, Kanta Jamil, Peter Kim Streatfield

**Affiliations:** International Centre for Diarrhoeal Disease Research, Bangladesh (icddr,b), 68 Shaheed Tajuddin Ahmed Sharani, Mohakhali, Dhaka, 1212 Bangladesh; United States Agency for International Development (USAID)/Bangladesh, Madani Avenue Baridhara, Dhaka, 1212 Bangladesh

**Keywords:** Adult female mortality, Causes of death, Verbal autopsy, BMMS, Bangladesh, NCD

## Abstract

**Background:**

Assessment of causes of death and changes in pattern of causes of death over time are needed for programmatic purposes. Limited national level data exist on the adult female causes of death in Bangladesh.

**Method:**

Using data from two nationally representation surveys, the 2001 and 2010 Bangladesh Maternal Mortality Surveys (BMMS), the paper examines the causes of adult female death, aged 15–49 years, and changes in the patterns of these deaths. In both surveys, all household deaths three years prior to the survey were identified. Adult female deaths were then followed by a verbal autopsy (VA) using the WHO structured questionnaire. Two physicians independently reviewed the VA forms to assign a cause of death using the ICD-10; in case of disagreement, a third physician made an independent review and assigned a cause of death.

**Results:**

The overall mortality rates for women aged 15–49 in 2001 and 2010 were 182 per 100,000 and 120 per 100,000 respectively. There is a shift in the pattern of causes of death during the period covered by the two surveys. In the 2001 survey, the main causes of death were maternal (20 %), followed by diseases of the circulatory system (15 %), malignancy (14 %) and infectious diseases (13 %). However, in the 2010 survey, malignancies were the leading cause (21 %), followed by diseases of the circulatory system (16 %), maternal causes (14 %) and infectious diseases (8 %). While maternal deaths remained the number one cause of death among 20–34 years old in both surveys, unnatural deaths were the main cause for teenage deaths, and malignancies were the main cause of death for older women. Although there is an increasing trend in the proportion of women who died in hospitals, in both surveys most women died at home (74 % in 2001 and 62 % in 2010).

**Conclusion:**

The shift in the pattern of causes of adult female deaths is in agreement with the overall change in the disease pattern from communicable to non-communicable diseases in Bangladesh. Suicide and other violent deaths as the primary cause of deaths among teenage girls demands specific interventions to prevent such premature deaths. Prevention of deaths due to non-communicable diseases should also be a priority.

## Background

Knowledge about causes of death is crucial for public health planning and resource allocation [1]. However, information on causes of death is limited in most developing countries. In these countries vital registration systems are either absent or poorly developed and lack quality information. Most deaths occur at home [2] and thus, information on causes of death is not available, except from very few areas that have comprehensive demographic surveillance system capturing information on deaths by cause, age and sex [3]. In order to get an estimate of cause specific mortality, these countries often rely on modeling exercises or on facility-based data, which is usually biased and incomplete for estimating causes of death.

Information on causes of death is especially important in view of the epidemiological transition that developing countries are experiencing. Although limited evidence exists, Bangladesh, a developing country in South Asia, is going through an epidemiological transition -- a shift in the mortality pattern from communicable diseases (CDs) to chronic diseases [4, 5]. Analysis of data from Matlab, a rural area in Bangladesh with a demographic surveillance system collecting information on causes of death since 1986, showed a substantial change in the mortality pattern from acute, infectious and parasitic diseases to non communicable, degenerative and chronic disease during the last 20 years [4].

In Bangladesh, there is a paucity of data on the causes of death at the population level. Although the Bangladesh Bureau of Statistics (BBS) maintains a nationally representative Sample Vital Registration System (SVRS) which records causes of death based on the information collected by a lay reporting system, there are reservations about the accuracy of the causes of death information from this system [6]. Studies have used verbal autopsy (VA) as a means to identify the causes of death among children and adult population. VA is a method of assessing probable causes of death based on an interview with the next of kin or other caregivers who were present at the time of death or who are knowledgeable about the events leading up to death. VA has been used previously in Bangladesh to ascertain causes of death including maternal deaths [7–9] and childhood deaths [10–19].

VA has increasingly been used to determine causes of death, especially in settings where deaths occur outside a hospital. Recent studies have suggested that VA can provide causes of death information that, at the population level, is similar to death certification in high-quality hospitals [20]. In this paper we examine changes in causes of adult female deaths using VA reporting on deaths from nationally representative sample of households.

## Methods

The present paper uses VA data from two nationally representative surveys, the 2001 and 2010 Bangladesh Maternal Mortality Surveys (BMMS), to examine the causes of adult female death aged 15–49 years, and the changes in the patterns of causes of death during the 9 year period between the two surveys. Both surveys were large and covered approximately 100,000 and 174,000 households respectively using nationally representative samples, sufficient to detect a 20 % decline in reduction of maternal mortality with 95 % confidence and 80 % power. In both surveys samples were selected through a three-stage sampling design [20, 21]. Both surveys used the same methodology to measure maternal mortality and assign the causes of maternal and non-maternal death over time. In each survey, the household questionnaire included questions on all household deaths that happened in about four years before the survey, and recorded the age, sex and date of death of each deceased person. Deaths of women of reproductive age (15–49) were subsequently followed up for VA using an adapted version of the 2007 version of the World Health Organization (WHO) questionnaire for adult deaths [22]. In the VA questionnaire information was collected on the signs and symptoms surrounding every death reported by the most knowledgeable person in the household. In the 2001 survey, 50 and in the 2010 survey, 60 female interviewers, each with at least a Bachelor’s degree, received a 7-days intensive training on the VA questionnaire before collecting information on the signs and symptoms surrounding every death reported by the most knowledgeable person in the household.

Cause of death was determined by independent review of the VA forms by two physicians. If they could not agree on the final cause of death, the case was then reviewed by a third physician. For a small number of cases where the three physicians agreed that the death was maternal but could not agree on a single cause of death, an expert committee of obstetricians was consulted to assign a cause of death. For each case, an underlying cause of death^1^ was coded using the International Classification of Diseases, 10^th^ Revision (ICD-10) classification. The underlying cause of death is grouped into eight categories: maternal; infectious diseases; malignancies; diseases of the circulatory system; suicide; other violent deaths; miscellaneous, and deaths for which cause of death was impossible to assign, or for which the reviewing physicians could not agree on a single cause of death. In this analysis, deaths that occurred in the three years preceding the two surveys among women aged 15–49 years are included.

Analysis included mortality rates and proportional mortality showing age specific patterns of causes of death, place of death and health seeking behavior before death. The mortality rate is calculated as the ratio of deaths in the three years preceding the survey to the person years lived by women aged 15–49 years (person years lived by specific age groups for age specific mortality) in the same period expressed per 100,000 women aged 15–49 years. Proportional mortality (%) was calculated by dividing the number of deaths attributed to a specific cause by the total number of deaths for which a VA was carried out. The difference between the two death rates in 2001 and 2010 was compared by calculating the 95 % confidence intervals (CI), and the difference between two proportions was compared by z-test for statistical significance at 80 % power and *p* < 0.05. Data were analyzed using SPSS version 17.0 and Microsoft Excel 2007. As the sample was not self-weighted household weights were used to get unbiased estimates at the national level.

This paper is a secondary analysis of existing de-identified data sets. Ethical approval for the BMMS 2001 and 2010 were obtained from the Ethical Review Committee (ERC) of the Bangladesh Medical Research Council (BMRC).

## Results

**Fig. 1:**
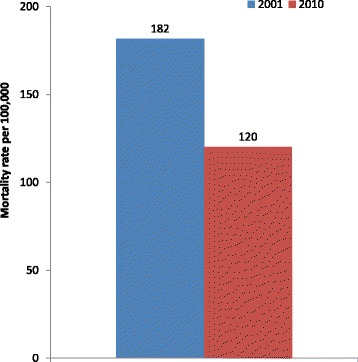
Mortality rates (per 100,000 women) among women aged 15–49 years: Bangladesh, 2001 and 2010

In the 2001 BMMS, a total of 696 deaths were reported among women 15–49 years in the three years prior to the survey. The number of such deaths was 749 in the 2010 survey. The overall mortality rates for this group of women in 2001 and 2010 were 182/100,000 (95 % CI 161-201/100,000) and 120/100,000 (95 % CI 107-132/100,000) respectively (Fig. 1).

Table 1 ranks the number of deaths in the three years preceding the two surveys by causes of death under eight categories. The highest numbers of deaths in the 2001 survey were due to maternal causes (140) and diseases of the circulatory system (103) whereas in the 2010 survey the highest numbers of deaths observed were due to malignancy (159), diseases of the circulatory system (122) and miscellaneous causes (122).

**Table 1:** Causes of death among women 15–49 years three years preceding the survey, Bangladesh, 2001 and 2010

	Causes of death	BMMS 2001 (*n* = 696)	Causes of death	BMMS 2010 (*n* = 746)
Maternal	APH/PPH	40	Indirect maternal death	37
	Hypertensive disorder of pregnancy	33	APH/PPH	35
	Unspecified/ undetermined	22	Hypertensive disorder of pregnancy	21
	Indirect maternal death	21	Obstructed labor	7
	Other maternal causes	10	Other maternal causes	5
	Abortion	7	Unspecified/ undetermined	1
	Obstructed labor	7	Abortion	1
Infectious diseases	Tuberculosis	31	Diarrohea	20
	Diarrohea	25	Tuberculosis	18
	Viral hepatitis	11	Typhoid and paratyphoid	9
	Other bacterial/viral/protozoal infection	11	Viral hepatitis	7
	Meningitis	5	Other bacterial/viral/protozoal infection	7
			HIV/AIDS	1
Malignancies	Cervix, uterus and other genital organs	19	Liver	30
	Leukemia	17	Other malignancies	28
	Liver	17	Cervix, uterus and other genital organs	26
	Lip, oral cavity and other digestive organs	12	Leukemia	23
	Other malignancies	12	Breast	20
	Stomach	7	Brain	10
	Breast	7	Lip, oral cavity and other digestive organs	9
	Brain	4	Stomach	6
	Respiratory organs	2	Anal canal	4
			Respiratory organs	3
Circulatory diseases	Cerebrovascular disease	54	Hypertensive heart disease	38
	Other form of cardiac disease	23	Cerebrovascular disease	32
	Ischemic heart disease	20	Other form of cardiac disease	29
	Rheumatic heart diseases	3	Rheumatic heart diseases	19
	Hypertensive heart disease	3	Ischemic heart disease	4
Suicide	Other chemicals	31	Hanging	26
	Pesticide	14	Other chemicals	18
	Hanging	13	Pesticide	16
	Others/unspecified	5	Smoke/fire	2
	Drowning	2	Others/unspecified	2
	Drug	1		
Other violent deaths	Others	9	Road traffic accident	12
	Smoke/fire	7	Drowning	12
	Snake bite	7	Fall	5
	Road traffic accident	5	Others	4
	Drowning	3	Smoke/fire	4
			Storm/lightening	3
			Iatrogenic	2
			Snake bite	1
Miscellaneous	Diseases of liver & gall bladder	27	Diabetes	23
	Other nervous system disorder	15	Others	20
	Chronic lower respiratory diseases	13	Diseases of liver & gall bladder	19
	Renal failure	12	Chronic lower respiratory diseases	16
	Gastric/peptic ulcer, appendicitis	11	Renal failure	11
	Others	8	Other diseases of respiratory system	9
	Diabetes	5	Other nervous system disorder	8
			Other diseases of urinary system	7
			Mental disorder	7
			Gastric/peptic ulcer, appendicitis	2
	Not classified	81	Not classified	67

Table 2 shows mortality rates of women by causes of death in the two surveys. In the 2001 survey the highest death rate was observed due to maternal causes (37/100,000), followed by circulatory diseases (26/100,000), malignancy (25/100,000) and infectious diseases (23/100,000), whereas in the 2010 survey, women had the highest mortality from malignancy (26/100,000) followed by death from circulatory diseases (20/100,000), maternal causes (17/100,000) and infectious diseases (17/100,000). When compared between the two surveys, the overall pattern of death rates shows a decreasing trend for almost all causes, except for deaths due to malignancy. However, none of these decreases was statistically significant except for death rates due to maternal (95 % CI 28-46/100,000 and 12-22/100,000 in 2001 and 2010 respectively) and infectious cause (95 % CI 16-30/100,000 and 7-14/100,000 in 2001 and 2010 respectively).

**Table 2:** Age-specific mortality rates (per 100,000 years of exposure) among women age 15–49 in the three years preceding the survey, by causes of death, Bangladesh 2001 and 2010

	Proportion of deaths																n	
	Maternal		Infectious		Malignancy		Circulatory disease		Suicide		Other violent causes		Miscellaneous		Not classified			
Age group	2001	2010	2001	2010	2001	2010	2001	2010	2001	2010	2001	2010	2001	2010	2001	2010	2001	2010
15–19	22.8	5.2	14.0	9.9	10.2	8.5	0.0	1.5	23.2	15.5	5.8	9.7	15.3	9.8	12.2	9.3	100	95
20–24	43.9	20.9	15.3	10.1	10.7	10.3	5.5	6.0	21.2	10.4	6.6	8.7	15.3	12.4	3.9	8.7	87	103
25–29	53.4	23.8	24.5	6.4	3.5	13.3	10.5	18.7	22.7	11.2	6.3	5.7	13.3	12.4	19.7	10.3	96	100
30–34	49.9	29.3	39.6	10.4	21.3	10.1	26.8	12.5	7.4	6.2	3.0	3.5	27.4	23.2	19.9	7.5	102	85
35–39	26.2	28.6	20.0	12.4	43.3	50.3	50.2	36.2	16.7	16.4	11.2	3.9	23.6	21.4	41.3	12.0	100	134
40–44	38.7	5.6	26.8	16.2	5.6	66.7	69.1	37.0	8.3	1.7	9.0	4.5	54.5	29.1	3.8	17.2	104	110
45–49	14.9	4.2	41.1	8.8	117.8	73.9	132.1	72.5	5.9	1.6	29.4	11.1	60.7	61.2	54.4	13.5	107	119
All (15–49)	36.7	17.0*	22.9	10.3*	25.4	25.6	26.3	19.7	17.5	10.3	8.0	7.0	23.9	19.7	21.1	10.5*	696	746

*p < 0.05

When the proportion of deaths by different causes were compared between the two surveys (Table 3), in the 2001 survey the main cause of death was maternal (20 %), followed by diseases of circulatory system (15 %), malignancy (14 %) and infectious diseases (13 %); in the 2010 survey, malignancy was the number one cause (21 %), followed by diseases of circulatory system (16 %), maternal causes (14 %) and infectious diseases (8 %). Between the two surveys, the relative share of deaths due to malignancy and diseases of the circulatory system increased significantly (*p* < 0.05) whereas the proportion of deaths due to maternal causes and infectious diseases decreased significantly (*p* < 0.05). The proportion of deaths due to suicide and other unnatural causes remained the same in both surveys.

**Table 3:** Proportional mortality among women age 15–49 in the three years preceding the survey, by causes of death, Bangladesh 2001 and 2010

	Proportion of deaths																n	
	Maternal		Infectious		Malignancy		Circulatory disease		Suicide		Other violent causes		Miscellaneous		Not classified			
Age group	2001	2010	2001	2010	2001	2010	2001	2010	2001	2010	2001	2010	2001	2010	2001	2010	2001	2010
15–19	22.0	7.4*	13.0	12.6	10.0	12.6	0.0	2.1	22.0	22.1	6.0	13.7	15.0	13.7	12.0	15.8	100	95
20–24	35.6	24.3	12.6	11.7	9.2	11.7	4.6	6.8	17.2	11.7	5.7	9.7	11.5	14.6	3.4	9.7	87	103
25–29	34.4	24.0	15.6	6.1*	3.1	13.1*	6.3	18.1*	14.6	11.0	4.2	6.0	8.3	12.0	13.5	10.0	96	100
30–34	25.5	27.1	20.6	10.6	10.8	9.4	13.7	11.8	3.9	7.1	2.0	3.5	13.7	23.5	9.8	7.1	102	85
35–39	11.0	17.2	8.0	6.7	18.0	26.9	22.0	19.4	7.0	9.0	5.0	2.2	11.0	11.9	18.0	6.7*	100	134
40–44	12.5	2.7*	8.7	9.1	19.2	37.3*	24.0	21.8	2.9	0.9	2.9	2.7	17.3	15.5	12.5	10.0	104	110
45–49	2.8	1.7	9.3	3.4	25.2	31.1	29.9	29.4	0.9	0.8	6.5	4.2	13.1	24.4*	12.1	5.0	107	119
All (15–49)	20.0	14.3*	12.5	8.3*	13.9	21.3*	14.8	16.4	9.5	8.6	4.6	5.8	12.9	16.4	11.8	9.0	696	746

*p < 0.05

As expected, cause specific death rates varied by different age groups (Table 2). The death rate from maternal causes increased beginning at age 20 and decreased after age 40. Death rates from circulatory diseases and malignancies increased sharply with age, whereas death rates from infectious diseases increased moderately with age. The suicide rate, on the other hand, was highest under the age of 30. Deaths due to other unnatural causes such as injuries, drowning, snakebites and other causes showed no clear age pattern. Both miscellaneous and unspecified and undetermined death rates increased moderately with age.

Distribution of deaths by different cause categories also varies by age groups (Table 3). While maternal deaths remained the number one cause of death among 20–34 years old in both surveys, unnatural deaths (suicide and other violent deaths) were the main causes for teenage deaths. In 2001, 28 % of teenage deaths were due to these causes, this figure increased in 2010 when 37 % of teenagers had unnatural deaths (*p* > 0.05). Unnatural deaths (suicide and other violent deaths) were the second leading cause of death for women aged 20–24 years after maternal deaths in both surveys; in 2001, 1 in every 5 women had suicidal deaths and an additional 7 % had other violent deaths. This figure decreased to 10 % for suicide and 9 % for other violent causes in 2010. A similar pattern of change in the causes of death is observed for 25–29 years age group for maternal and unnatural deaths. Diseases of the circulatory system and malignancies were the two leading causes of death among older women aged 35–49 years. These two diseases together accounted for 45 % of total deaths in 2001 and 50 % of total deaths in 2010 in this age group. In 2001, diseases of the circulatory system were the primary cause of death in this age group, followed by malignancies whereas malignancies ranked as the main cause in 2010 followed by diseases of the circulatory system. Women died from different types of malignancies and there was little variation in the type of malignancies between the two surveys. In 2001, among women who died from malignancies, the distribution by type malignancy was: cancers of the gastrointestinal tract, including liver (37 %), blood and lymphatic system (19 %), reproductive cancer including cancers of the reproductive organs including breast and cervical cancer (28 %) and other cancers (18 %). In 2010, women died from cancers of the gastrointestinal tract, including liver (30 %), cancers of blood and lymphatic system (28 %), reproductive cancers including cancer breast and cervical cancers (29 %) and other cancers (13 %). Under the miscellaneous category, diabetes, hepatitis and chronic respiratory diseases were the main causes of death in both surveys.

Although there is an increasing trend in the proportion of women who die in health facilities, the majority of women still die at home. In the 2010 survey, 38 % women died in a health facility compared to 26 % in 2001 (46 % increase; *p* < 0.05) as shown in Table 4. The increase in facility deaths was observed in all categories of deaths except in unnatural deaths.

**Table 4:** Proportion of deaths that happened in health facilities by causes of death, Bangladesh, 2001 and 2010

Causes of death	BMMS 2001 (*n* = 696)	BMMS 2010 (*n* = 746)	% increase/decrease between two surveys
Maternal	29	45*	57
Infectious diseases	15	21*	38
Malignancy	13	14	3
Circulatory diseases	15	35*	142
Suicide	35	39	12
Other violent causes	19	14*	- 26
Miscellaneous	7	23*	245
Not specified	5	12*	142
All	26	38*	46

*p < 0.05

Care seeking before death also improved (Table 5). In the 2010 survey, overall 78 % of women sought care from a health facility or pharmacy before death whereas this figure was 63 % in the 2001 survey (24 % increase; *p* < 0.05). While this increase was observed among all death categories except unnatural deaths, the increase was highest for deaths due to circulatory diseases (68 % increase; *p* < 0.05), followed by miscellaneous causes (44 %; *p* < 0.05) and maternal causes (29 %; *p* < .05). In both 2001 and 2010, public facilities were the most prominent source of care. However, in the 9 years between the two surveys, there has been a substantial increase in seeking care from private health facilities (increased from 12 to 46 %).

**Table 5:** Proportion of deceased who sought care before death, Bangladesh, 2001 and 2010

Causes of death	Place(s) of seeking care before death													
	Any Facility/ pharmacy		Public facility		NGO facility		Private facility		Pharmacies^1^		Home		n	
	2001	2010	2001	2010	2001	2010	2001	2010	2001	2010	2001	2010	2001	2010
Maternal	55	71*	39	50*	2	5	16	26*	15	10	44	41	139	107
Infectious diseases	72	71	52	40	6	6	5	37*	30	22	40	38	87	62
Malignancy	90	98*	73	72	2	2	27	75*	49	36*	35	21*	97	159
Circulatory diseases	50	84*	38	56	2	0	11	53*	20	29	42	21*	103	122
Suicide	52	52	41	44	0	0	3	5	13	7	14	6	66	64
Other violent causes	52	35*	47	33*	4	1	4	13	9	3*	39	22*	32	43
Miscellaneous	59	85*	41	32*	0	3	13	56*	32	21*	45	31*	90	122
Not specified	70	75*	41	42	7	0	5	47*	45	34	37	27	82	67
All	63	78*	46	55	3	2	12	46*	28	23	38	26	696	746

*p < 0.05

^1^ Include qualified and unqualified health providers in pharmacies

## Discussion

This paper presents, for the first time ever, provides national estimates of causes of adult female deaths and provides a unique opportunity to to examine changes in the patterns of causes of death over time. Overall, there has been a major shift in the pattern of causes of death over the 9 year period. While deaths due to maternal and infectious origins are significant contributors of adult female deaths, deaths due to non-communicable diseases (NCDs) such as circulatory diseases, malignancies, diabetes and other chronic conditions are on the rise. Unnatural deaths including deaths due to suicides are also a major killer.

While this study provides national level estimates of cause specific female mortality and the causes of death, the findings should be interpreted with caution, given the difficulties in assigning an exact cause of death using the VA method. Verbal autopsy, as a method of ascertaining causes of death has increasingly been recommended, especially when death occurs outside of health facilities and without the presence of a medically trained person to assign a cause of death [20, 23–25]. However, the validity of using VA to assess the causes of death depends on the extent and the accuracy of signs, symptoms and circumstantial evidence collected through the VA questionnaire and then on the review of this information and assignment of an appropriate cause of death by a skilled reviewer. The validity of using VA to ascertain causes of death may also vary by age group of the deceased and the cause of death [26].

We used an adapted version of the WHO questionnaire for adult deaths [27], have employed highly trained interviewers to conduct the interviews and, finally, have engaged trained physicians with previous experience in reviewing the VA questionnaires and assigning ICD-10 codes for causes of death. We also used similar methodology to collect and interpret data from both surveys so that the results are highly comparable. Even though we are unable to compare our findings with a gold standard -- death certificates, or hospital records, there is evidence from validation studies done in other settings where physician reviewed VA results were compared to the gold standard and found to have high diagnostic accuracy for diseases like cardiovascular diseases and malignancies [28] and other causes of adult death [29].

A methodological challenge in assigning an exact cause of death using the VA method is also reflected in the proportion of deaths that are being categorized as “not-classified”. In both surveys, about 10 % of deaths were categorized as “not-classified” for which it was either impossible to assign a cause of death on the basis of information collected through VA or the reviewing physicians could not agree to a single cause of death. If an appropriate diagnosis could be made for these cases, the cause specific mortality reported here might be affected to some extent. However, previous studies using similar verbal autopsy method as a means to assess the causes of death have reported either higher or similar figures for “unknown” or “unclassified” categories of death [7, 8].

Our findings are in agreement with other studies documenting NCDs as a major cause of adult death including female deaths in Bangladeshi [4, 7, 20, 30, 31]. Labrique et al.’s study conducted in a cohort of rural Bangladeshi women found NCDs as the leading cause of death among women of reproductive age (48 %), followed by pregnancy related causes (22 %), infectious diseases (17 %) and injury (9 %). Circulatory diseases accounted for the largest proportion of NCD deaths (40 %), followed by cancer (15 %) [30]. Similarly, Alam et al.’s study conducted in another rural area of Bangladesh also found that 65 % of all female deaths aged 15–59 years were due to NCDs, followed by deaths due to communicable diseases (15 %), pregnancy related deaths (10 %) and injury related deaths (8 %) [7].

Similar to many other developing countries, Bangladesh is going through a shift in the disease and mortality patterns from acute infectious and parasitic diseases to non-communicable, chronic diseases. The 2010 update of Global Burden of Disease Study estimated that between 1990 and 2010, proportion of deaths due to NCDs in Bangladesh increased from 34 to 60 %, whereas contribution of communicable diseases (including maternal, neonatal, and nutritional disorders) nearly halved (56 % to 30 %) and the proportion of injury remained around 10 % (NCDs) [32]. Given the prominence of NCDs in the mortality statistics and their potential impact on the socioeconomic development of the country, the government of Bangladesh has identified NCDs as a priority and has taken steps to strengthen activities for the prevention and treatment of these diseases. While the prevention efforts should be focused on identifying and modifying risk factors through practicing healthy life-style, early detection and treatment are also important to decrease the long term impact of these diseases.

Our results indicate that a substantial proportion of female deaths occurred due to intentional and unintentional harm, with suicide being the leading cause of death among female teenagers in both surveys. While limited information exists on the extent and the causes of suicide among the Bangladeshi population, a recent study conducted to understand the epidemiology of suicide in Bangladesh found that suicide is the fourth injury related deaths in Bangladesh and the leading cause of injury related deaths among adolescents aged 10–19 years, with slightly higher risk of suicide among females compared to males [33]. In this study the overall suicide rate among adult females was found to be 8 per 100,000 which is lower than the overall suicide rate we found in our study (23 per 100,000 in 2001 and 16 per 100,000 in 2010). Another study conducted in Matlab, a rural area of Bangladesh, found an average annual death rate due to suicide among women 15–44 years old of 13 per 100,000 in 1982–1998 [34], which is also lower than the present study.

Because of methodological differences we cannot compare previous study findings with our study findings. However, suicide is an issue of growing concern in Bangladesh with some areas reporting 37 % of all deaths of women aged 15–44 years attributable to suicide [35]. Longitudinal data collected from different demographic surveillance sites of icddr,b suggest that the percentage of deaths attributable to suicide among women, particularly among young women of reproductive age, is increasing [35].

We are also unable to explain the underlying causes of this high level of unnatural deaths. However, other studies have identified a number of circumstances leading to suicides among young girls and women including emotional and verbal abuse by husbands and other family members, financial hardship in the family, childlessness and rejection of marriage offer or unacceptable partner [34]. Prevention of suicides is thus challenging and requires multifaceted approaches and adaptation of strategies to locally relevant cultural factors. A detailed investigation of these deaths, along with identification of their determinants, is needed before any programmatic actions can be taken to reduce such untimely deaths.

Using BMMS data, this paper presents the cause and age specific adult female deaths at the national level in two surveys, almost a decade apart, and comparing them between the two surveys. The sample size for the two surveys was calculated to have sufficient statistical confidence and power to estimate the national MMR and calculate the changes in MMR over the 9 year period between these surveys but not to estimate cause specific mortality or age and cause-specific mortality or to compare them between the two surveys. However, the results are strongly indicative of the decreasing trend for death rates for some diseases (maternal and infectious diseases).

There is an increasing trend in seeking care before death and in the proportion of deaths that happened in health facilities. The increase in seeking care is more marked for conditions like circulatory diseases and diseases like diabetes, chronic respiratory conditions etc. The majority of women visited public facilities for seeking care. Given that more than 70 % of the country’s population live in rural areas where the first point of care usually is public facilities that are primarily designed to provide maternal and child health care services, whether these facilities are equipped to manage the increasing number of NCD cases needs to be explored.

## Conclusion

Bangladesh has made remarkable progress in the health and development sector in the last three decades. Bangladesh is one of the nine countries that are on track to achieve the Millennium Development Goal (MDG) 5 of reducing maternal mortality. This was made possible through improvements in the health sector as well as in non-health sectors. However, much still needs to be done to prevent untimely deaths of women. Our study provides a solid basis in support of the argument that Bangladesh is going through an epidemiological transition and NCDs are significant contributors to adult female deaths. Given the prominence of NCDs in the mortality statistics and their potential impact on the socioeconomic development of the country, the government of Bangladesh has identified NCDs as a priority area and has taken steps to strengthen activities for the prevention and treatment of these conditions. However, only 4.5 % of the national health budget in the Health Population and Nutrition Sector Development Program (HPNDSP 2011–16) has been allocated for these diseases. The Government efforts should be strengthened further to take this trend into account and to increase the proportion of the health budget for NCDs, and to use evidence-based information on how to tackle these conditions. While prevention efforts should be focused on identifying and modifying risk factors through practicing healthy life-style, early detection and treatment are also important to decrease the long term impact of these diseases.

## Abbreviations

BBS: Bangladesh Bureau of Statistics
BMMS: Bangladesh Maternal Mortality Surveys
CDs: Communicable diseases
ICD-10: The International Classification of Diseases- 10^th^ revision
HPNDSP: Health Population and Nutrition Sector Development Program
MDG: Millennium Development Goal
MMR: Maternal mortality ratios
NCDs: Non-communicable diseases
SVRS: Sample Vital Registration System
VA: Verbal autopsy
WHO: World Health Organization

## Footnotes

1 The underlying cause of death is defined as the disease or injury which initiated the chain of morbid events leading directly to death, or the accident/act which produced the fatal injury.
